# Delineating the impact of machine learning elements in pre-microRNA detection

**DOI:** 10.7717/peerj.3131

**Published:** 2017-03-29

**Authors:** Müşerref Duygu Saçar Demirci, Jens Allmer

**Affiliations:** 1 Department of Molecular Biology and Genetics, Izmir Institute of Technology, Urla, Izmir, Turkey; 2 Bionia Incorporated, IZTEKGEB A8, Urla, Izmir, Turkey

**Keywords:** MicroRNA, Machine learning, Feature selection, Negative dataset, ML strategy, *Ab initio* pre-miRNA detection

## Abstract

Gene regulation modulates RNA expression via transcription factors. Post-transcriptional gene regulation in turn influences the amount of protein product through, for example, microRNAs (miRNAs). Experimental establishment of miRNAs and their effects is complicated and even futile when aiming to establish the entirety of miRNA target interactions. Therefore, computational approaches have been proposed. Many such tools rely on machine learning (ML) which involves example selection, feature extraction, model training, algorithm selection, and parameter optimization. Different ML algorithms have been used for model training on various example sets, more than 1,000 features describing pre-miRNAs have been proposed and different training and testing schemes have been used for model establishment. For pre-miRNA detection, negative examples cannot easily be established causing a problem for two class classification algorithms. There is also no consensus on what ML approach works best and, therefore, we set forth and established the impact of the different parts involved in ML on model performance. Furthermore, we established two new negative datasets and analyzed the impact of using them for training and testing. It was our aim to attach an order of importance to the parts involved in ML for pre-miRNA detection, but instead we found that all parts are intricately connected and their contributions cannot be easily untangled leading us to suggest that when attempting ML-based pre-miRNA detection many scenarios need to be explored.

## Introduction

An organism’s phenotype largely depends on the expression levels of its genes and proteins. Gene regulation determines the amount of RNA and its alternative forms that are expressed in a cell and this step is influenced by transcription factors. Some of the transcripts are subsequently translated into protein. This step is modulated by post-transcriptional regulators like small non-coding RNAs such as MicroRNAs (miRNAs) (Erson-Bensan, 2014). In metazoan miRNAs stem from hairpin-like structures which are cut from nascent RNAs via Drosha (Bartel, Lee & Feinbaum, 2004). These structures are then exported into the cytoplasm where Dicer processes them and aids in the incorporation of one single stranded part of them into RISC which finally facilitates the binding to mRNA and, thereby, the regulation of its translation efficiency (Erson-Bensan, 2014). This pathway requires both miRNA and target mRNA to be expressed at the same time. Expression is, however, often bound to extrinsic stimuli or stresses (Berezikov, Cuppen & Plasterk, 2006). Therefore, it is hard to investigate miRNA regulation experimentally and even harder to try to establish all miRNA target interactions for any higher eukaryotic organism.

This difficulty led to the development of computational approaches for the detection of miRNAs and their targets (Allmer & Yousef, 2012; Saçar & Allmer, 2014). Two approaches have been employed, one making use of evolutionary information while the other is based in machine learning (ML). For *ab initio* miRNA detection, using ML, a number of strategies have been proposed (Saçar & Allmer, 2014). Differences among ML approaches are in the positive and negative example sets, where the latter has no quality guarantees (Yousef et al., 2008). For the parameterization of pre-miRNAs several hundred features have been proposed and including normalization methods, there are more than one thousand features available to parameterize a hairpin (Saçar & Allmer, 2013a). Machine learning algorithms like support vector machine (SVM), naïve Bayes (NB), random forest (RF), and many more have been employed to establish models for pre-miRNA detection (Ng & Mishra, 2007; Jiang et al., 2007) with RF being the currently most popular ML algorithm according to a Google Scholar search (Fig. S1). Models have been trained using different schemes in respect to the split between training and testing sets, cross validation methodology, and number of models trained. Finally, some studies report tuning of the learning algorithm’s parameters (Yousef et al., 2008). Thus, the overall ML system consists of roughly five modules (Saçar & Allmer, 2014):

(1) Training and testing data selection
(2) Feature selection
(3) Machine learning algorithm selection
(4) Training scheme establishment
(5) Machine learning algorithm parameter tuning

The dictum in ML is ‘garbage in garbage out’ (Yang, 2010) which may be interpreted such that the selection of training and testing data (1) is the most important among the tasks that lead to model establishment. Parameterization (2) is considered to be as important as data selection, while the other parts (3–5) are attributed with lesser impact on model performance.

In this study we developed two novel negative dataset, one of which was intended to be difficult to solve (ColShuf). Whereas the other should be relatively easier (RowShuf) to solve. Finally, we used a third one (pseudo) which has been widely employed in pre-miRNA detection. Additionally, we used eight positive datasets one from miRTarBase (Hsu et al., 2011), two consisting of differentially filtered mouse data from miRBase (Griffiths-Jones et al., 2006) and five from MirGeneDB (Fromm et al., 2015). We created three different feature sets based on speed and variance. For training, we used one main scheme while another was tested and did not tune ML parameters so we did not fully account for (4) and (5) in this study while we employed six ML algorithms for model establishment. We abstained from tuning ML parameters (5) in this work since we previously showed that some parameter tuning affects ML algorithms differently (Saçar Demirci, Toprak & Allmer, 2016). Our results indicate that there was no obvious trend to attach more effect to any one part of the ML system (1–5). In conclusion it became clear, that multiple ML algorithms need to be tried since not all of them may be equally applicable in respect to selected features and selected datasets. The exceptional good performance of RF is a bit suspicious but in line with the expectations attached to a modern ensemble classifier.

## Materials and Methods

Pre-miRNA detection is mostly performed using classification-based machine learning approaches. As described above, we suggest five major influences on the effectiveness of established models. In order to analyze the impact of these five parts to machine learning, different datasets were acquired or created, several *ad hoc* feature selection methods were applied and multiple machine learning algorithms were used under an established training regime.

### Datasets

In order to perform machine learning, high quality training data is essential and both positive and negative examples need to be provided when using two class classification. Given such data machine learning models can be trained which can be applied to unknown data. This is in order to test the influence of module (1) which shows the impact of training and testing data selection. For this we obtained and/or created a number of datasets of varying levels of quality to ensure a broad spectrum of test results. This allows the assessment of the system at different performance levels.

### Positive data

*Mus musculus* hairpin sequences and their related information like reads per million (RPM) values were obtained from miRBase (release 21). The RPM > 100 dataset included only the hairpins with RPM values equal or greater to 100 (380 hairpins). The RPM < 100 dataset included only the hairpins with RPM values between 0 and 100 (813 hairpins). The MiRTarBase (release 6.0) dataset included the mouse hairpins with experimentally supported targets (weak support entries were not included; 229 hairpins).

In addition, 523 *Homo sapiens*, 395 *Mus musculus*, 229 *Gallus gallus* and 287 *Danio rerio* hairpins obtained from MirGeneDB v1.1 (http://mirgenedb.org/) were used as positive datasets individually and collectively.

### Negative data

Since true negative pre-miRNA data is not available, pseudo negative data needs to be generated. The ‘pseudo’ dataset (8,492 hairpins) is a popular negative dataset used in various studies (Jiang et al., 2007; Chen, Wang & Liu, 2016; Lopes, Schliep & De Carvalho, 2014) on the detection of pre-miRNAs and it was downloaded from Ng & Mishra (2007). No other negative dataset has been used by more than one study on pre-miRNA detection. Therefore, we present two novel negative datasets which are based on data shuffling.

The column shuffling (ColShuf) dataset was created by shuffling the values of all positive datasets on a per column basis. Thus the distribution of the data for each features remained unchanged which entails that the dataset should be difficult to differentiate from positive data. The row shuffling dataset (RowShuf) was created by shuffling the values of the positive datasets on a per row basis. Therefore, the value distribution for each feature was largely changed and the resulting dataset should be easily distinguished from the positive data. Consequently, three negative datasets were created with ColShuf expected to be most difficult, followed by pseudo and finally RowShuf which should be easiest to solve. Datasets are available upon request.

### Features

Hundreds of features for the parameterization of pre-miRNAs have been described, but we showed that 50 features are sufficient for successful application of machine learning to pre-miRNA detection (Saçar & Allmer, 2013a). Calculating such features is possible using existing web servers (http://jlab.iyte.edu.tr/software/izmir and Yones et al., 2015). To investigate the impact of feature selection, three different feature selection strategies were followed to create feature sets of varying effectiveness to enable testing the system at different performance levels. The low variance filtered feature set (LowVarFS) was created by using the “Low Variance Filter” node in KNIME (Berthold et al., 2008) in an attempt to select features which will display low performance. The variance upper bound was set to 0.000001 and all features displaying a larger variance were removed. The other two feature sets were selected according to the computational time it takes to calculate each feature. The FastFS feature set represents features which can be calculated quickly and from previous experiments it was known that such features can help distinguishing between positive and negative class to a degree. The SlowFS feature set consists of the 50 features requiring the longest computational time. Time required calculating each feature was based on the analysis of 1,881 human hairpin sequences. Features with high computational cost are for example probability-based or employ complex structural calculations and are, thus, deemed to be more performant than others. Consequently, three feature sets of varying expected success rate were generated. The expectation was that SlowFs should lead to the best models followed by FastFS and finally LowVarFS. The intuition here is that simple sequence based features are quickly calculated but not very discriminative while structure based features are computationally more involved but better able to detect pre-miRNAs. For each feature set 50 features were selected (Table S1).

### Learning

For machine learning a host of classifiers are available and many of them have been used to detect pre-miRNAs.

**Figure 1:**
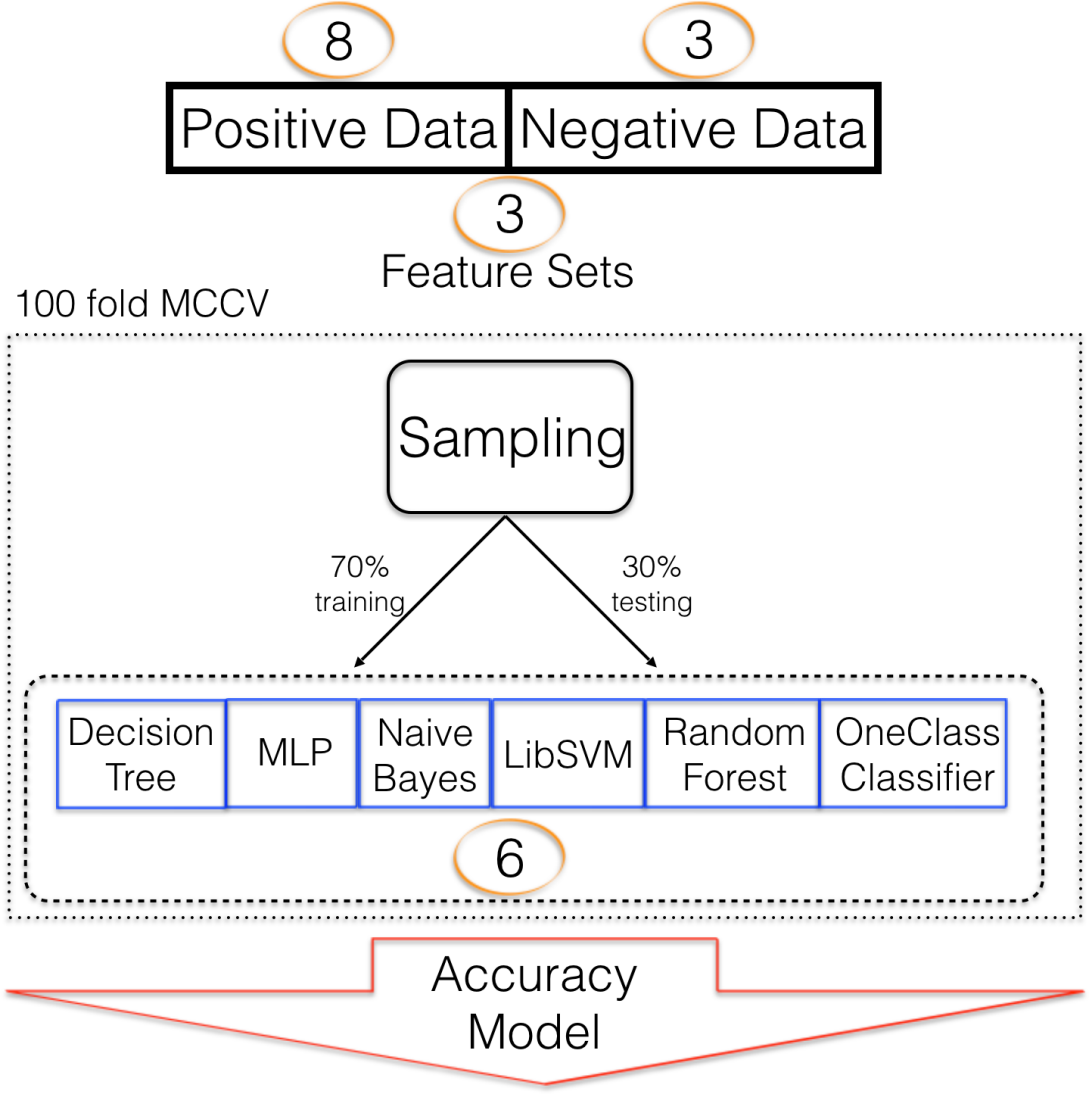
Machine learning strategy. Machine learning strategy employed to establish models describing pre-miRNAs for mouse. Number of alternative sets are written in circles e.g., 3 different feature sets were used.

Six different classifiers were used for model training. Decision trees (Quinlan, 1986) (DT) are constructed based on how features separate training data into the target and negative class. Multiple such decisions are built into a decision tree which can then be used for predictive analyses. We have previously used DT to detect pre-miRNAs (Saçar Demirci, Bağcı & Allmer, 2016). Random forest (RF) is based on DT but many not fully induced decision trees are created and used as an ensemble for predictive analyses (Tin kam Ho, 0000). Random forest has been used in pre-miRNA detection (Lopes, Schliep & De Carvalho, 2014)). Multi-layer perceptron (MLP) was previously applied to pre-miRNA detection by Gudyś et al. (2013). They also used naïve Bayes (NB) and support vector machine (SVM), a popular classification algorithm in machine learning (Vapnik, 1995), for pre-miRNA detection. While two class classification with the previously mentioned machine learning strategies is possible, in the absence of a well-defined negative class, one class classification can be useful and it has been shown that it can be applied to pre-miRNA detection (Yousef et al., 2008). Here we employ KNIME (Berthold et al., 2008) for machine learning and use all algorithms with their default settings. Since it wasn’t possible to attach a prediction to the ML algorithms’ performance before the experiments, a variety of them were used. In a previous study, RF performed best for us, though (Saçar Demirci, Bağcı & Allmer, 2016). In addition to the selection of the machine learning algorithm, a strategy to train the algorithm needs to be selected. A large number of approaches exist ranging from leave-n out cross validation to *k*-fold cross validation (CV). Selection of such strategies is dependent on data availability and has been assessed before (Varma & Simon, 2006; Kohavi, 1995). For this study we found that 100 fold Monte Carlo cross validation (MCCV, Xu & Liang, 2001) was most successful compared to *k*-fold CV (*k* = 10, Figs. S2 and S4). The split of data into training and testing fractions is also influenced by data availability and we were able to choose a 70 to 30 learning to testing ratio. Other studies used 90/10 training/testing which, in our opinion, leads to overestimation of the ML success. Between positive and negative data a one to one ratio was maintained to avoid class imbalance which has a large impact on model performance (Saçar & Allmer, 2013a). The complete training scheme is summarized in Fig. 1.

## Results and Discussion

Machine learning can be broken down into five coarse components (see above) which together establish an ML strategy (Fig. 1). We investigated the effect of various training and testing datasets, different sets of selected features and six ML algorithms on classifier performance. It is the aim of this study to investigate the impact of constituting modules on overall performance and, therefore, datasets, feature selection methods and selected ML algorithms are of varying quality. We did not attempt to optimize for performance, but rather investigated the relative impact of ML modules on classification outcome.

**Figure 2:**
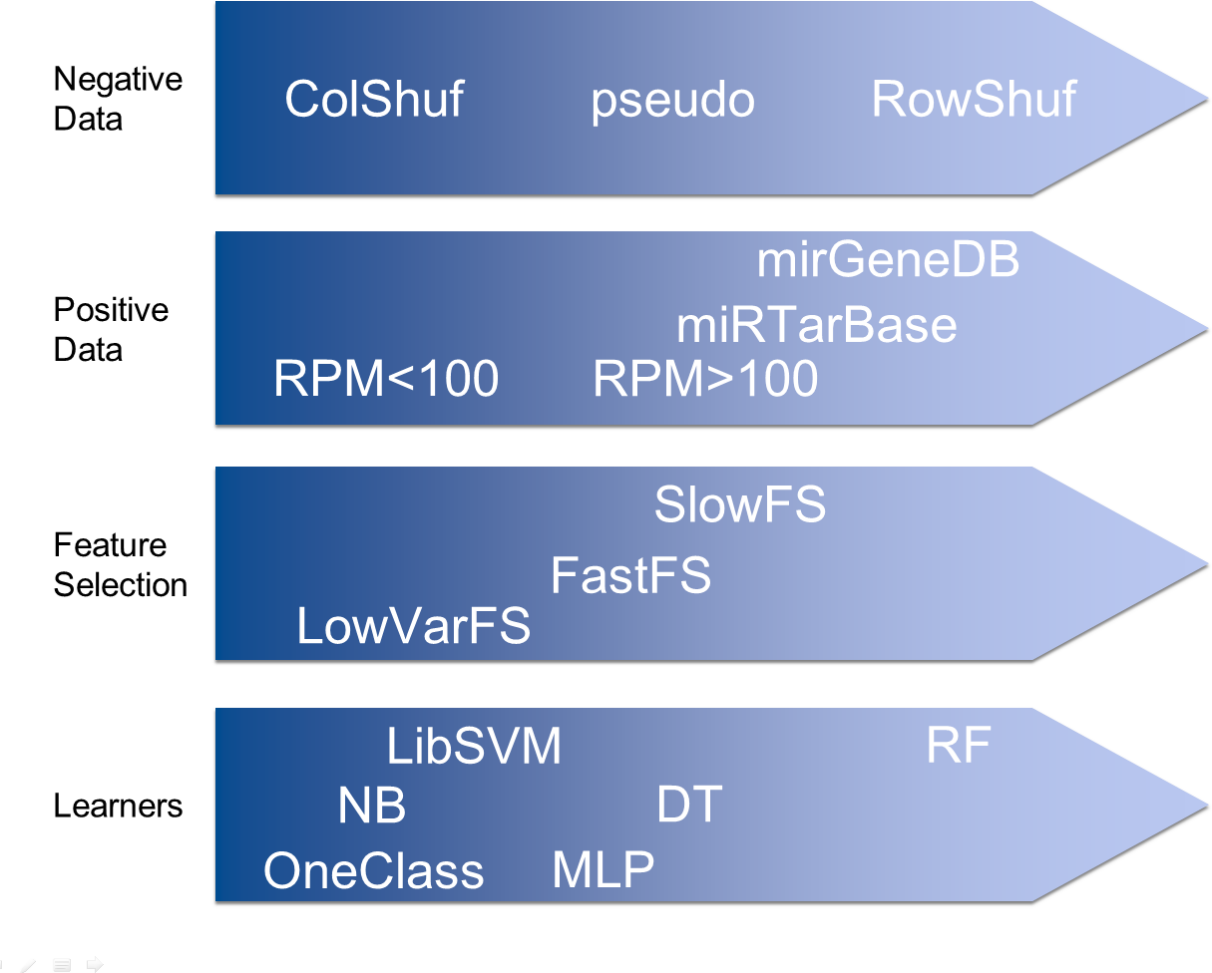
Effect of machine learning elements on model performance. Effects of various parameters on the overall classification performance. Items more to the left are either more difficult to be solved (negative data, positive data, and feature set) or it is more difficult for them to reach high performance (learners).

As a training/testing strategy 100 fold Monte Carlo cross validation was used (Fig. 1). We performed this training/testing scheme for all 432 combinations and collected 43,200 performance measures in that way (File S2). First we sorted the results decreasingly by accuracy and assigned a rank to each combination. Then we summed the ranks for the three different negative datasets and it was confirmed that overall the RowShuf dataset was easier to solve than the pseudo one which in turn was easier to solve than the ColShuf dataset. RowShuf had a more than 2 fold lower rank sum compared to ColShuf and pseudo had a one fold lower rank sum (Fig. 2). Using the same approach for positive data showed that all three datasets are of similar difficulty collecting almost equal rank sums with RPM < 100 being slightly (∼9%) more difficult than miRTarBase and about 8% more difficult than RPM > 100 (Fig. 2). A similar result was observed for the three feature selection methods. Employing FastFS made it easier to create good ML models compared to LowVarFS. Using SlowFS made it about 9% easier to establish efficient models than FastFS (Fig. 2). The ML algorithms used in this study, random forest, decision tree, multi-layer perceptron, support vector machine (LibSVM), naïve Bayes, and OneClass performed quite different for the 432 ML strategies. RF was about 3 times and DT about 2 times more successful than OCC. MLP was about 1.5 times and LibSVM about 1.2 times more successful than OCC. Finally, NB was about 10% more successful than OneClass according to rank sum (Fig. 2).

### Effect of training examples on classifier performance

**Figure 3:**
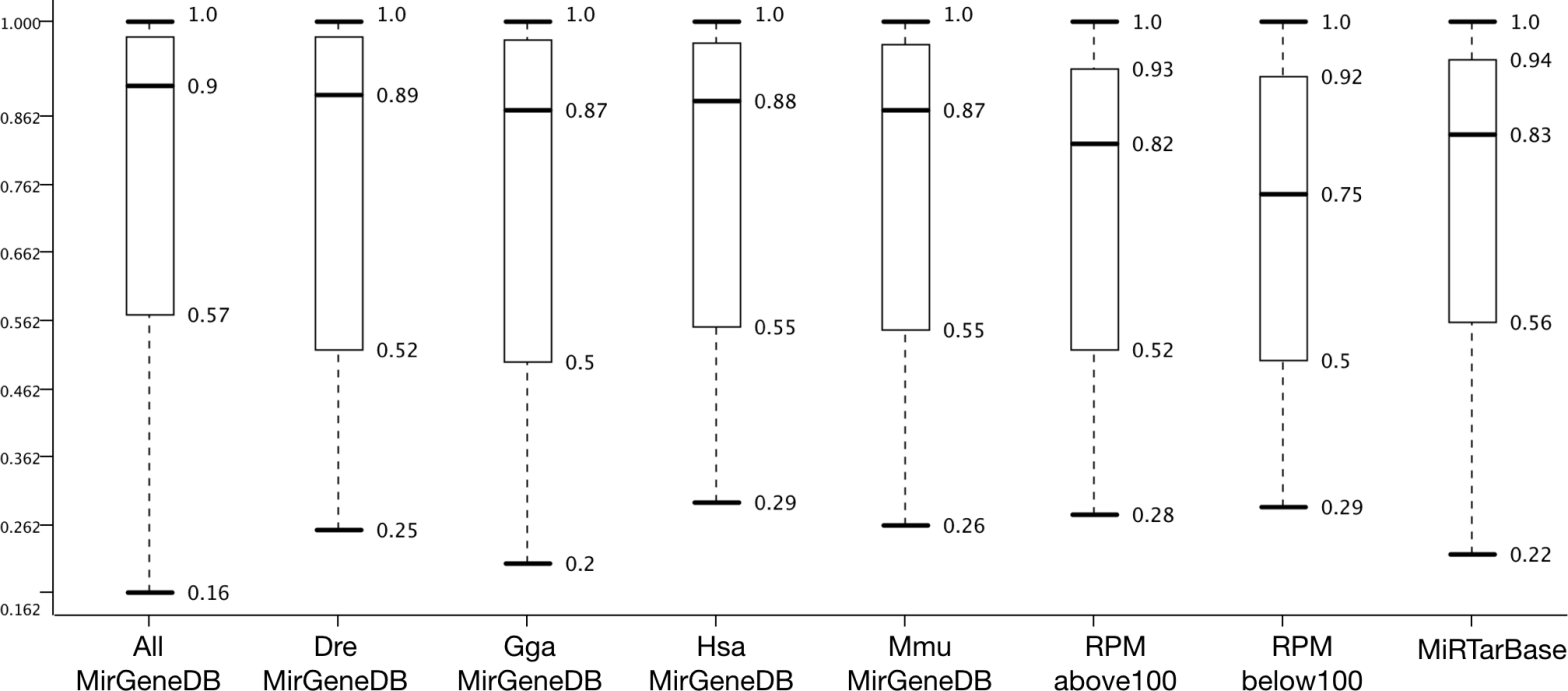
Dataset effect on model performance. Accuracy distribution at 100 fold MCCV for the eight positive datasets employed in this study (see Fig. 4 for negative datasets). All other variables were summarized (e.g., six learners).

The rank assessment above does not take into account the accuracy achieved during testing the classifiers with the 30% examples held back for testing. For 10-fold CV, which represents a 90-10 training testing scheme with 10% examples available for testing (Files S1, S3; and Figs. S2 and S4), slightly better accuracy distribution were achieved when compared to 100-fold MCCV. Accuracy may not be the best measure to compare classifier performance, but when looking at an accuracy distribution this judgement may change (Fig. 3).

Figure 3 shows that AllMirGeneDB leads to the best accuracy distribution among 432 cases and 100 repetitions. It has the highest median (0.90) and the second smallest inter quartile range (IQR: 0.41; 0,38 for miRTarBase) and the second highest third quartile (0.975; 0.977 for DreMirGeneDB). In our previous works we found that *Mus musculus* examples in miRBase may be contaminated with non-pre-miRNAs. When grouping these examples into ones with less and more than 100 reads per million (RPM) the accuracy distribution changes and for the examples with lower expression (<100 RPM) the minimum, first quartile, median and IQR are all worse than for RPM > 100 and miRTarBase or MmuMirGeneDB results. The latter dataset performs even better than the RPMabove100 filtered version of *M. musculus* from miRBase for all criteria highlighting the data quality in MirGeneDB. Overall, MirGeneDB data led to better performing models than miRTarBase. Interestingly, using all data available on MirGeneDB leads to the best performance with a median 0.07 points and a third quartile 0.035 better than for miRTarBase. That MirGeneDB may still contain a number of non-miRNA entries can be seen from the minimum which is the lowest for all datasets (0.16).

Although 10-fold CV lead to slightly better results than 100-fold MCCV (Files S2, S3, and Figs. S2–S4), we show the MCCV results here. This is because we prefer a larger amount of test examples over slight improvements in accuracy distribution which may be misleading due to lower relative amount of test examples in 10-fold CV. Additionally, the aim here is to look at differences not attempting to establish the best possible model.

Negative examples cannot be established experimentally, since it is not possible to co-express all putative miRNAs with all there possible target mRNAs. Therefore, all negative data is arbitrary, but we have previously used the pseudo dataset with success (Saçar & Allmer, 2013b).

**Figure 4:**
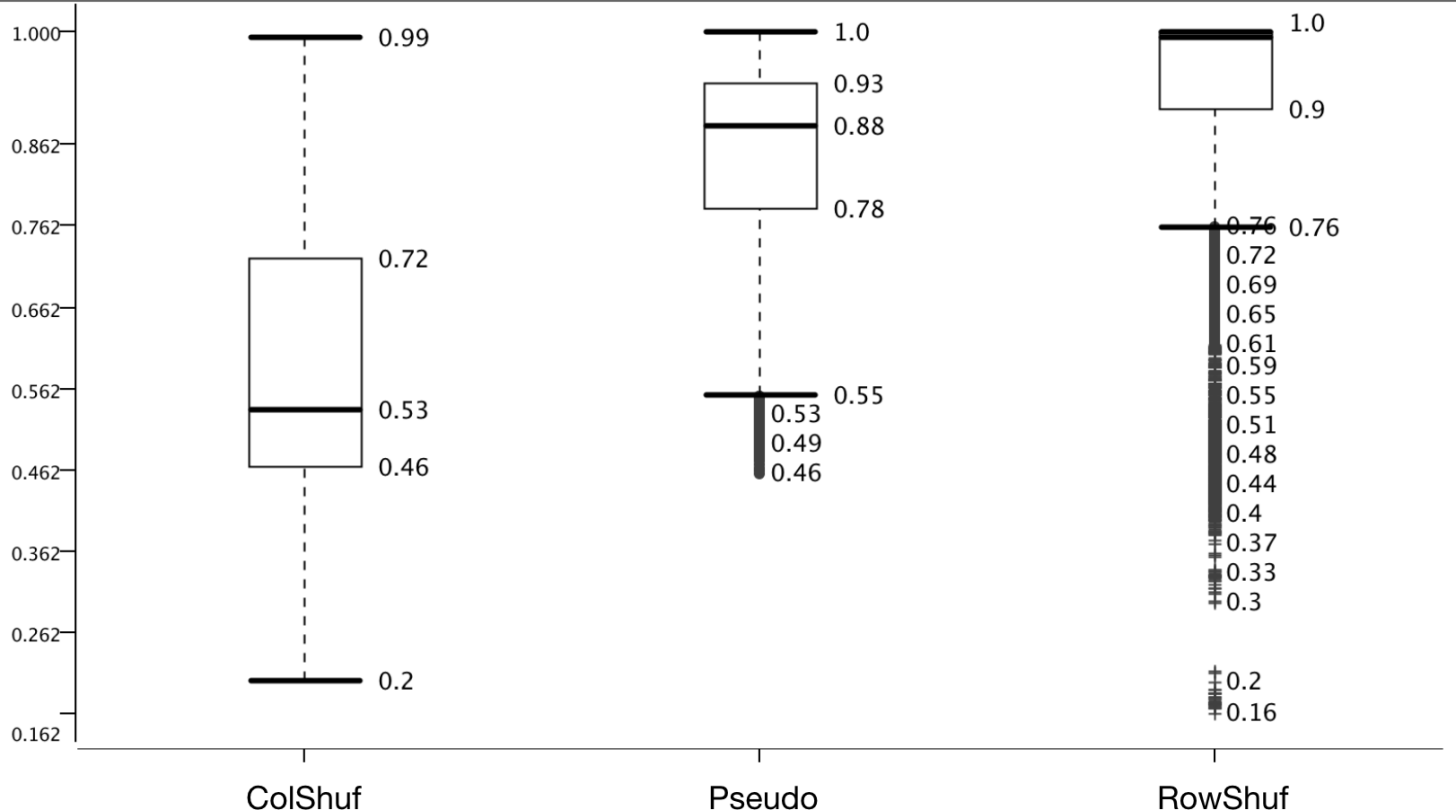
Effect of negative data selection on model performance. Accuracy distribution at 100 fold MCCV for the eight positive datasets. All other variables were summarized (e.g., six learners).

Two new negative datasets are proposed here; one which is easy to solve (RowShuf) since it shuffles the matrix row-wise and therefore breaks the feature vectors and one which should be difficult to solve (ColShuf) since it preserves the calculations on a feature vector basis and should, therefore, be quite similar to the original positive data. The accuracy distribution confirms this expectation and ColShuf shows worst (with a median around random guessing) while RowShuf displays the best accuracy distribution with a medium of 1.0 (Fig. 4). The approach used here to generate negative data from positive data has not been used in the field of pre-miRNA detection and while RowShuf is too simple to solve (similar to random sequence generation Saçar & Allmer, 2013b), ColShuf is quite difficult to solve and can be used as an additional challenge in training and testing of classifiers for pre-miRNA detection in future studies. We did not use other negative datasets like randomly generated sequences or sequences of a specific type (e.g., exonic ones). Because the former does not deviate much from RowShuf in performance and the latter is partially included in pseudo.

### Effect of feature selection on classifier performance

**Figure 5:**
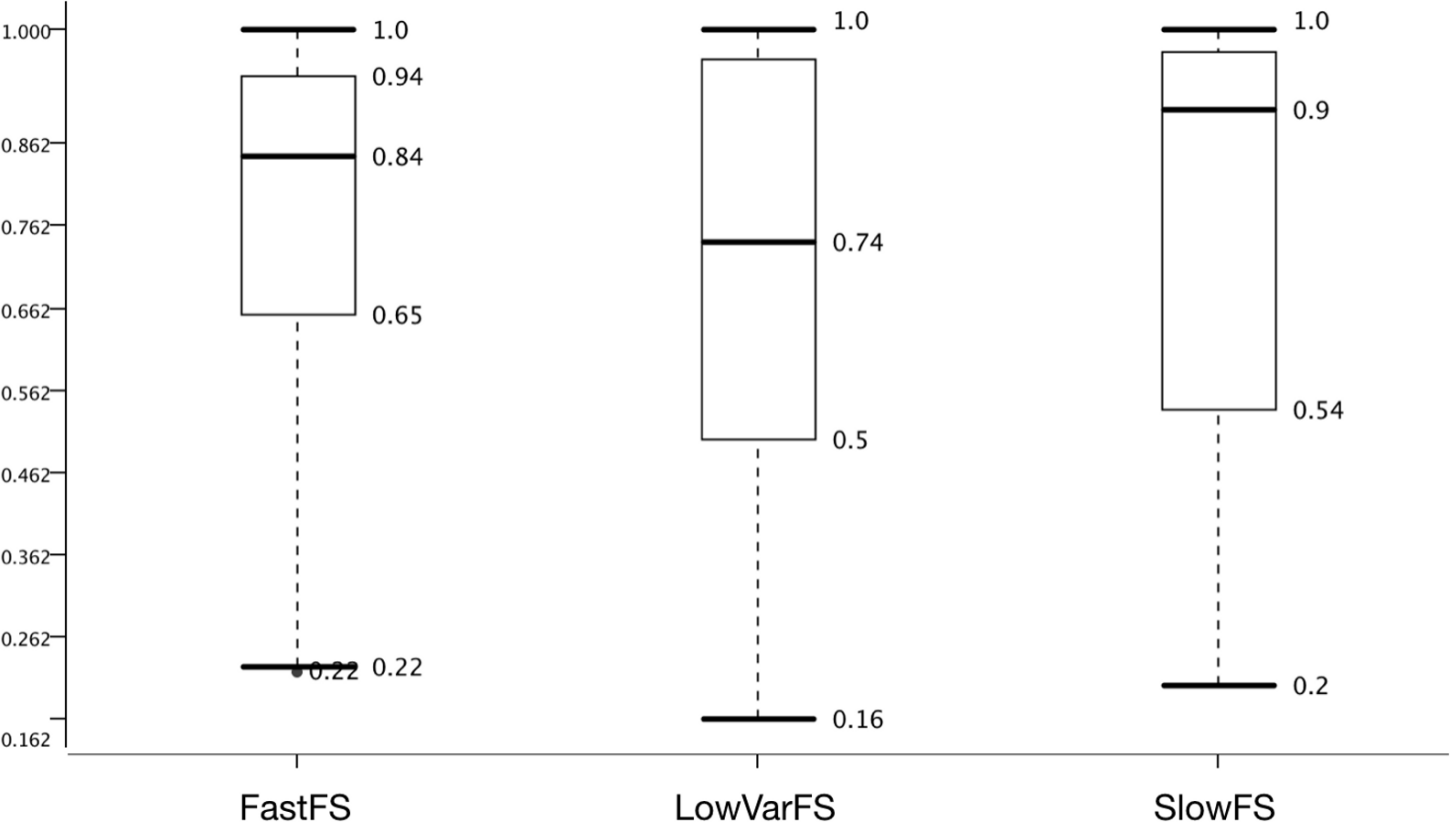
Effect of feature selection on model performance. Accuracy distribution based on Feature sets with the datasets and learners summarized.

Many pre-miRNA detection studies employing ML have been proposed and one difference among them is using various selected feature sets (Ng & Mishra, 2007; Bentwich et al., 2005; Ding, Zhou & Guan, 2010). Unfortunately, feature selection is NP-hard (Amaldi & Kann, 1998) and, therefore, the best feature set has not been determined. Three feature selection methods were tried with the expectation of selecting the 50 features with the lowest variance (LowVarFS) to be the least effective (Fig. 5).

Additionally, the 50 features that take the longest computational time (SlowFS) were expected to perform best. Figure 4 confirms this expectation and it is seen that the median is highest for SlowFS. It also becomes clear that such simple feature selection, as performed here, has its limitation which is seen in the IQRs which was small for FastFS and large for LowVarFS. Many other feature selection methods have been employed before (Paul, Magdon-Ismail & Drineas, 2015; Guyon et al., 2002; Ahsen et al., 2012; Lorena, Carvalho & Lorena, 2015; Xuan et al., 2011; Hall et al., 2009) including filter, wrapper, and embedded methods. Here we use simple *ad hoc* strategies in an attempt to create feature sets which vary in performance. Compared to the negative datasets this was less successful since the medians are within 0.26 points of each other whereas the difference is 0.47 for negative datasets. For the positive datasets on which we had little influence, the difference between best and worst performing ones is only 0.15 points.

### Effect of ML algorithm on classifier performance

For the detection of pre-miRNAs a number of models have been developed using different ML algorithms. The chosen ML algorithm may have an impact on the model performance even when keeping all other variables constant and, therefore, we selected six ML algorithms to analyze the impact on classifier performance. These algorithms were chosen from different domains and they are not variants of each other as DT and C4.5 are. However, random forest is an ensemble method which is based on decision trees and, therefore, represents a variant. The general success of ensemble methods warrants the use of RF, though.

Random forest performed best in respect to all measures like IQR and median; followed by DT. OneClass performed worst which is likely due to feature selection which has a strong impact on OCC (Yousef et al., 2016; Yousef, Allmer & Khalifa, 2016). While the median of OCC represents random guessing, RF is only 0.05 points away from full accuracy. Thereby, the range between worst models and best models is 0.45 points which is similar to the difference among negative datasets. In summary, we were able to setup the overall system such that in model and negative data performance a large variance was achieved. The variance in feature selection and positive data was not as large, but sufficient for this study.

**Figure 6:**
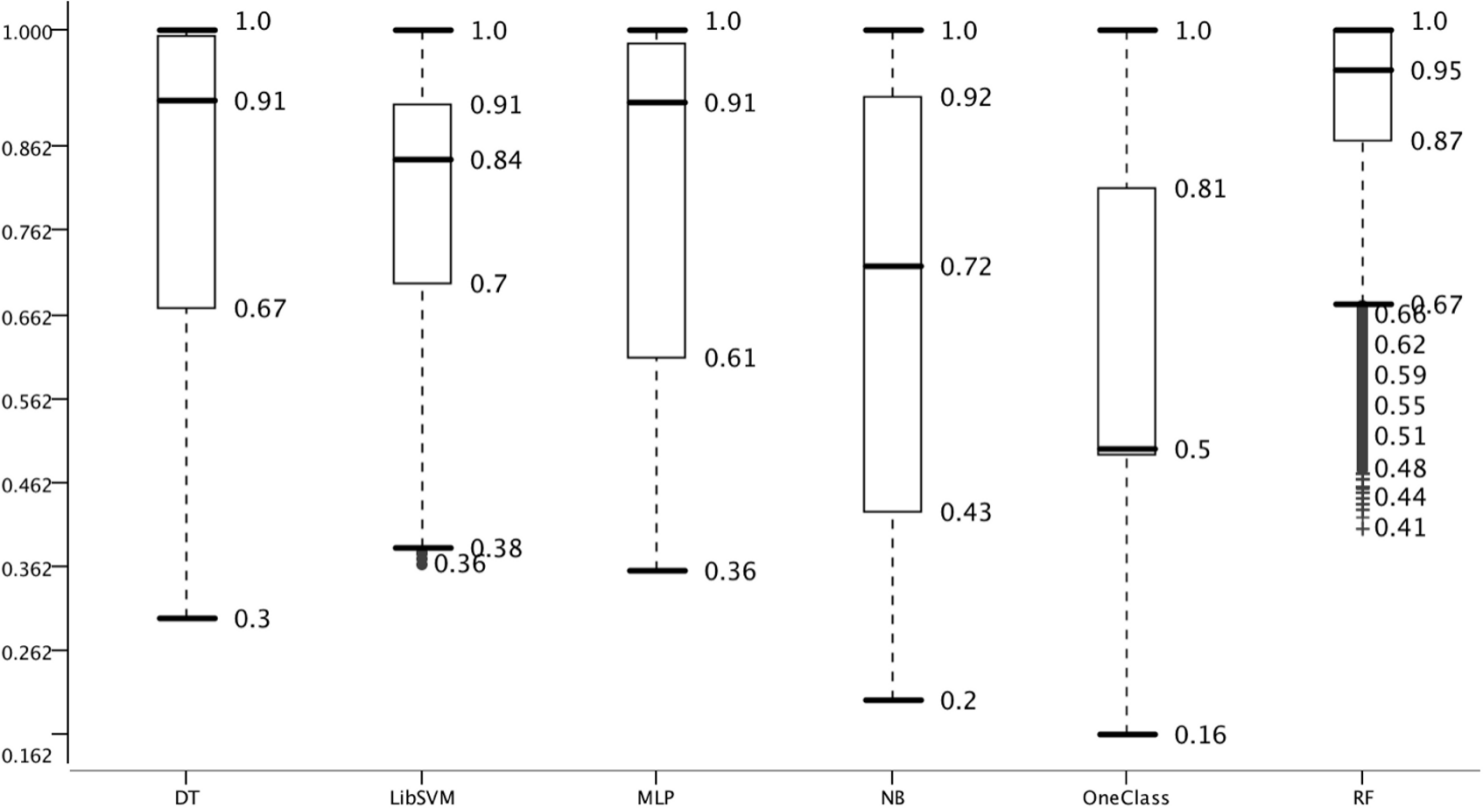
Effect of choice of machine learning algorithm on model performance. Accuracy distribution of the six classifiers used for 100 MCCV. All other parameters are summarized.

**Figure 7:**
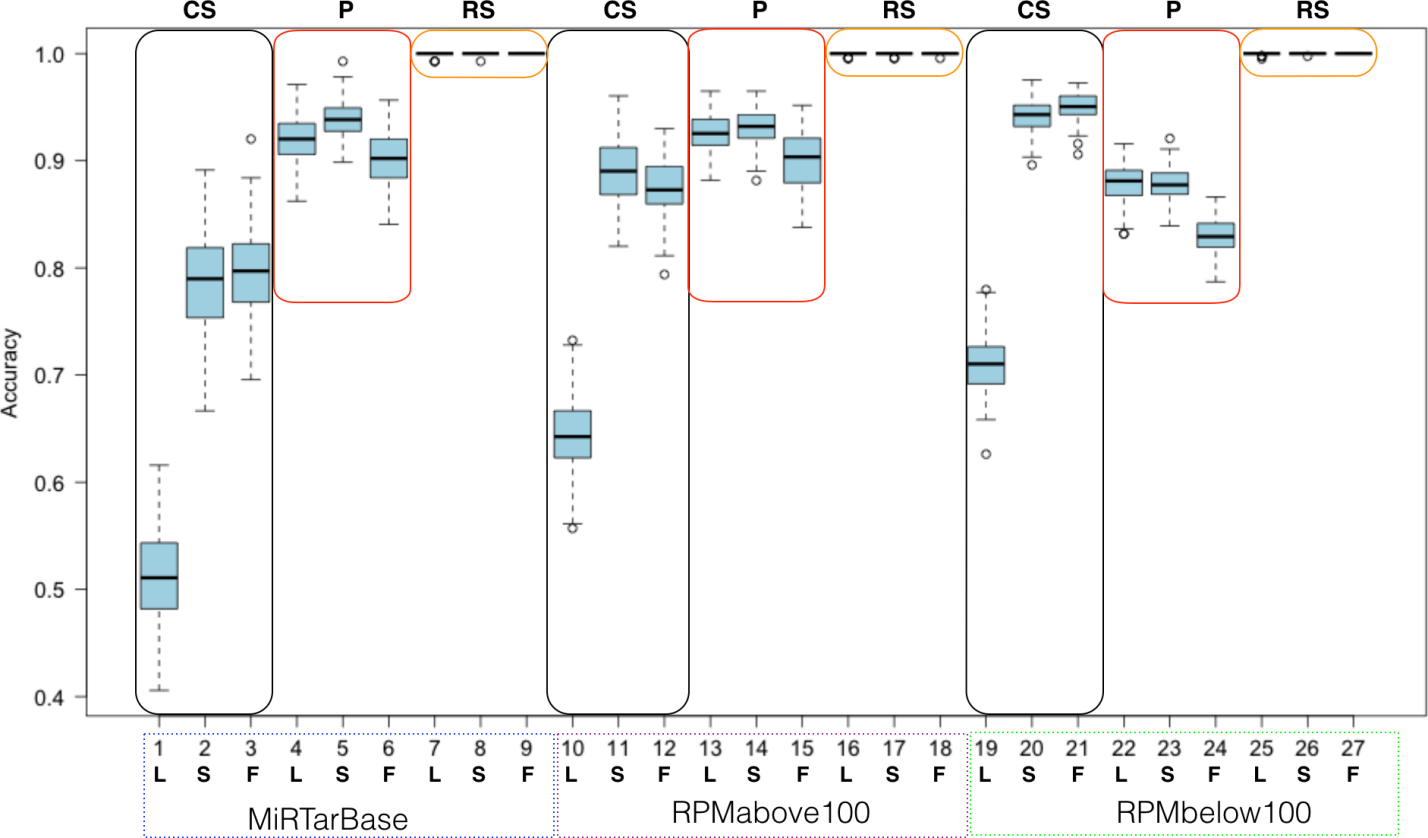
Summarized random forest performance. RF performance based on all combinations of parameters. L, S, F stand for LowVarFS, SlowFS and FastFS, respectively. The three negative datasets ColShuf (CS), pseudo (P), and RowShuf (RS) are listed across the top naming their associated rectangles. Positive datasets are indicated below the x-axis. Each box plot refers to 100 fold MCCV.

The overall system is difficult to present including all variables. Therefore, and due to the exceptional performance of RF (Fig. 6), we analyzed the results from the viewpoint of RF (Fig. 7).

When analyzed from the perspective of one classification algorithm the part of ML that becomes most important is the dataset. For example, the RowShuf dataset always performs best. Also, there are large differences in respect to positive data while the accuracy distribution in respect to feature selection is smaller except for the expectedly bad performance of LowVarFS. Similar results as in Fig. 7 were achieved using the MirGeneDB datasets (Fig. S3). It is obvious from Fig. 7 that the RowShuf dataset is too simple and can easily be solved under all circumstances. On the other hand, the combination of LowVarFS and ColShuf leads to worst performance close to random guessing. Interestingly, this is most pronounced for the highest quality positive dataset MiRTarBase and least for the lowest quality dataset RPMbelow100. The pseudo dataset shows a stable performance under all conditions and performance varies only slightly. Conversely to ColShuf, pseudo leads to better performance in combination with higher quality positive data. This analysis was from the viewpoint of RF but other ML algorithms may lead to different outcomes. Therefore, we performed the same analysis for NB (medium performance) and OCC (lowest performance) to investigate whether the same trends exist (Figs. S2 and S3). The general trends are supporting our findings for RF with the expected decrease in overall performance (Figs. S5–S8). All three ML approaches show lower performances when LowVarFS is used (Fig. 7 and Figs. S2–S8). In the case of OCC, it seems that most of the models are making random guessing with performance values around 0.5. For NB, LowVarFS and ColShuf combination seems the most challenging case independent of the positive dataset (Figs. S5–S6) but unlike OCC, NB performances shows reversed labeling instead of random guessing, meaning that 0.4 or lower accuracy value would lead to a performance 0.6 if the class decision was simply reversed.

## Conclusion

The application of machine learning to biological data has become important and in pre-miRNA analysis it has become indispensable (Jiang et al., 2007; Lopes, Schliep & De Carvalho, 2014; Gudyś et al., 2013; Ding, Zhou & Guan, 2010; Bentwich, 2008; Batuwita & Palade, 2009; Van der Burgt et al., 2009; Gao et al., 2013). ML is a system which is influenced by different choices that can be made, for example, the selected training and testing datasets, feature selection, and the choice of classification algorithm. These choices impact the overall model performance. Three negative and positive datasets, three feature selection methods, and six ML algorithms were used to establish ML models using 100 fold MCCV and 10 fold CV. We used the default settings of ML algorithms within KNIME and did not attempt to optimize them. This may be an explanation for the difference in performance among algorithms which lead to almost 3 fold differences when looking at the overall ranks achieved in the 43,200 established models (Fig. 2). When analyzing the results from the perspective of using one particular ML algorithm with optimized settings, for simplicity we chose RF which performed very well, the largest impact on classification performance stems from the choice of data. A note of caution, it is important to choose a dataset which is not trivial to solve and which is also not too hard to be solved and then iteratively attempt to solve more difficult datasets (Allmer, 2012). Here we showed that the RowShuf dataset would be too easy to solve and the ColShuf dataset too difficult. While this is a general observation from our results, there are occasional outliers where an ML algorithm leads to a good model using low quality training and testing data.

Thus, we conclude that there is no means to predict the performance of an ML strategy and it is important to try multiple classifiers, feature selection methods, and example datasets.

##  Supplemental Information

10.7717/peerj.3131/supp-1 File S1 Figures S1–S8 and Table S1 This file contains a collection of supplementary figures (S1–S8) and the Table S1 which lists the selected features.

10.7717/peerj.3131/supp-2 File S2 All established model performances Collection of all established model performances for 100 fold MCCV (43200).

10.7717/peerj.3131/supp-3 File S3 All established model performances The recorded model performances for 10-fold cross validation (720).

10.7717/peerj.3131/supp-4 File S4 Negative Datasets Spreadsheet containing all synthetic negative datasets produced in this study.

## Additional Information and Declarations

### Data Availability

The following information was supplied regarding data availability: The raw data has been supplied as a supplementary file (File S2).
