# Supplementary material for: Delineating the impact of machine learning elements in pre-microRNA detection
Source: PeerJ. 2017 Mar 29;5:e3131. doi: 10.7717/peerj.3131 (PMC5374968; doi:10.7717/peerj.3131)
Supplement: File S1 — This file contains a collection of supplementary figures (S1–S8) and the Table S1 which lists the selected features. [file peerj-05-3131-s001.docx]

**Delineating the impact of machine learning elements in pre-microRNA detection**

**Müşerref Duygu Saçar Demirci^1^ and Jens Allmer^1,2^**

^1^Molecular Biology and Genetics, Izmir Institute of Technology, Urla, Izmir, Turkey
^2^Bionia Incorporated, IZTEKGEB A8, Urla, Izmir, Turkey

**Abstract**

Gene regulation modulates RNA expression via transcription factors. Post-transcriptional gene regulation in turn influences the amount of protein product through, for example, microRNAs (miRNAs). Experimental establishment of miRNAs and their effects is complicated and even futile when aiming to establish the entirety of miRNA target interactions. Therefore, computational approaches have been proposed. Many such tools rely on machine learning (ML) which involves example selection, feature extraction, model training, algorithm selection, and parameter optimization. Different ML algorithms have been used for model training on various example sets, more than 1000 features describing pre-miRNAs have been proposed and different training and testing schemes have been used for model establishment. For pre-miRNA detection, negative examples cannot easily be established causing a problem for two class classification algorithms. There is also no consensus on what ML approach works best and, therefore, we set forth and established the impact of the different parts involved in ML on model performance. Furthermore, we established two new negative datasets and analyzed the impact of using them for training and testing. It was our aim to attach an order of importance to the parts involved in ML for pre-miRNA detection, but instead we found that all parts are intricately connected and their contributions cannot be easily untangled leading us to suggest that when attempting ML-based pre-miRNA detection many scenarios need to be explored.

Figure S1: Trends in ML algorithm applications on miRNA analysis based on Google Scholar results (December 2016).


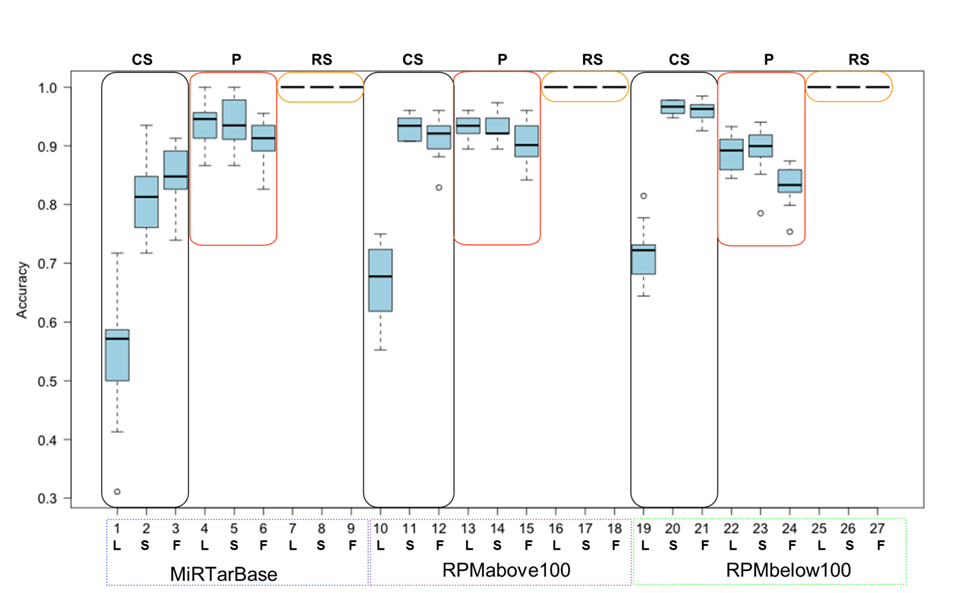


Figure S2: RF performance based on all combinations of parameters. L, S, F stand for LowVarFS, SlowFS and FastFS, while CS, P and RS refer to ColShuf, Pseudo and RowShuf, respectively. Rectangles indicate the three negative datasets while positive datasets are indicated under the x-axis. Each box plot refers to 10 fold CV calculations.


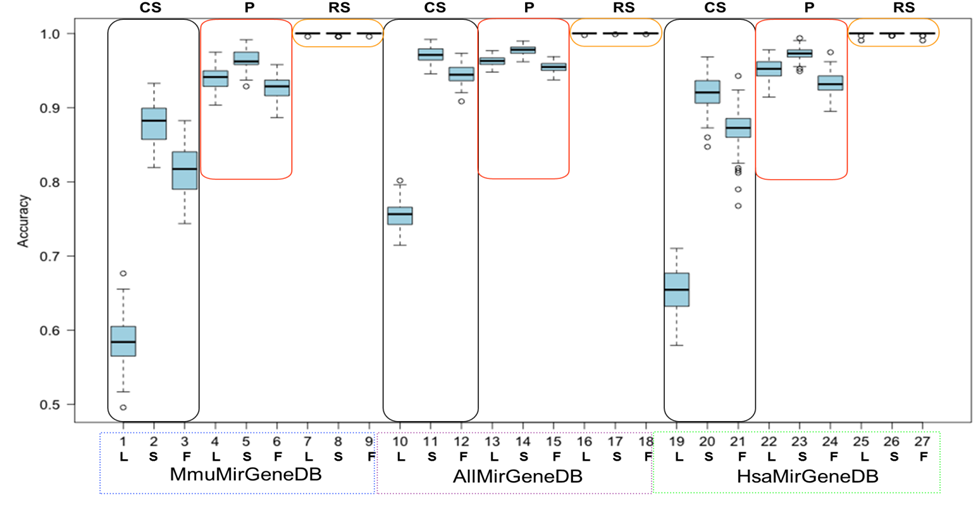


Figure S3: RF performance based on all combinations of parameters. L, S, F stand for LowVarFS, SlowFS and FastFS, while CS, P and RS refer to ColShuf, Pseudo and RowShuf, respectively. Rectangles indicate the three negative datasets while positive datasets are indicated under the x-axis. Each box plot refers to 100fold MCCV calculations.


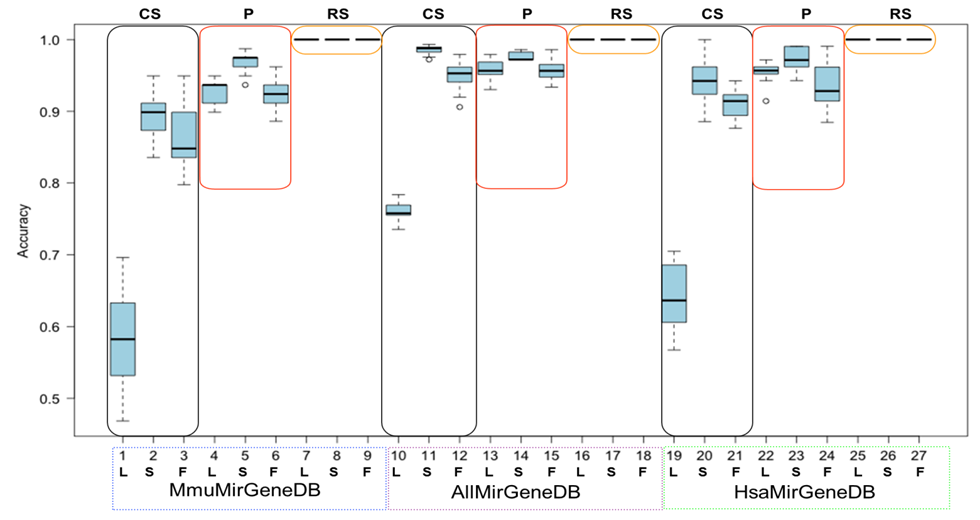


Figure S4: RF performance based on all combinations of parameters. L, S, F stand for LowVarFS, SlowFS and FastFS, while CS, P and RS refer to ColShuf, Pseudo and RowShuf, respectively. Rectangles indicate the three negative datasets while positive datasets are indicated under the x-axis. Each box plot refers to 10fold CV calculations.


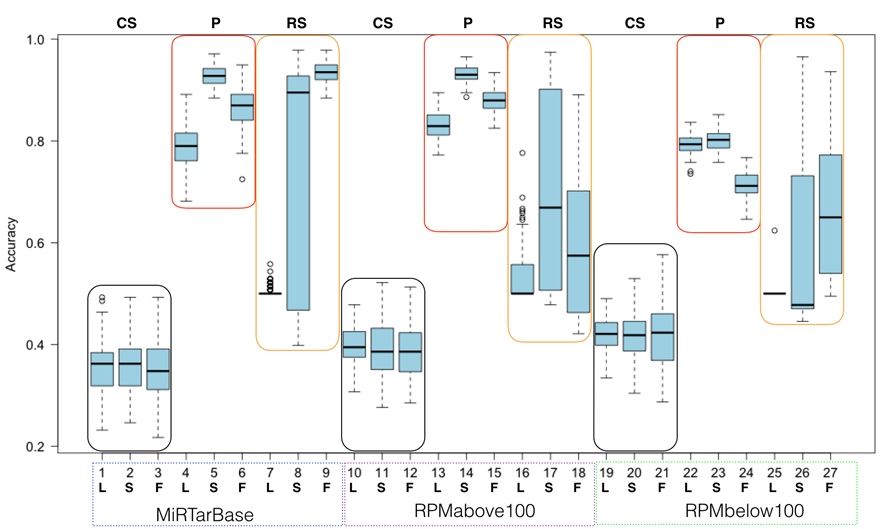


Figure S5: NB performance based on all combinations of parameters. L, S, F stand for LowVarFS, SlowFS and FastFS, while CS, P and RS refer to ColShuf, Pseudo and RowShuf, respectively. Rectangles indicate the three negative datasets while positive datasets are indicated under the x-axis. Each box plot refers to 100fold MCCV calculations.


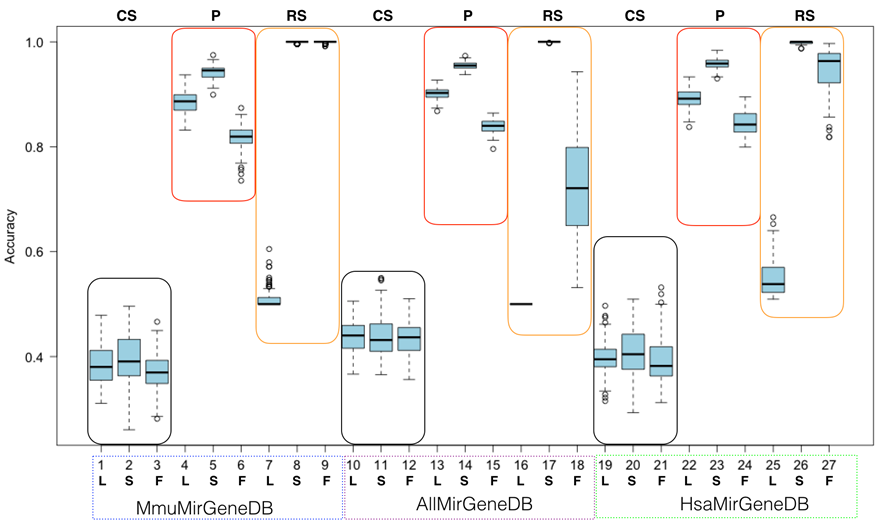


Figure S6: NB performance based on all combinations of parameters. L, S, F stand for LowVarFS, SlowFS and FastFS, while CS, P and RS refer to ColShuf, Pseudo and RowShuf, respectively. Rectangles indicate the three negative datasets while positive datasets are indicated under the x-axis. Each box plot refers to 100fold MCCV calculations.


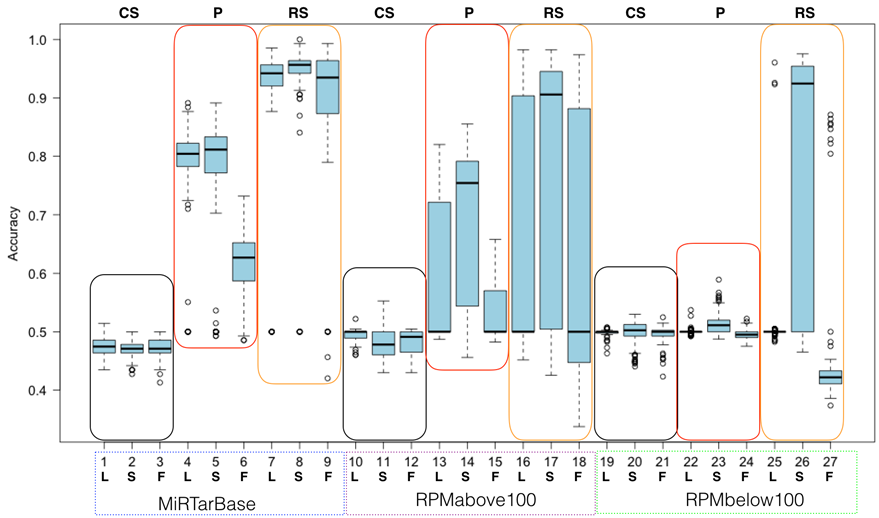


Figure S7: OCC performance based on all combinations of parameters. L, S, F stand for LowVarFS, SlowFS and FastFS, while CS, P and RS refer to ColShuf, Pseudo and RowShuf, respectively. Rectangles indicate the three negative datasets while positive datasets are indicated under the x-axis. Each box plot refers to 100fold MCCV calculations.


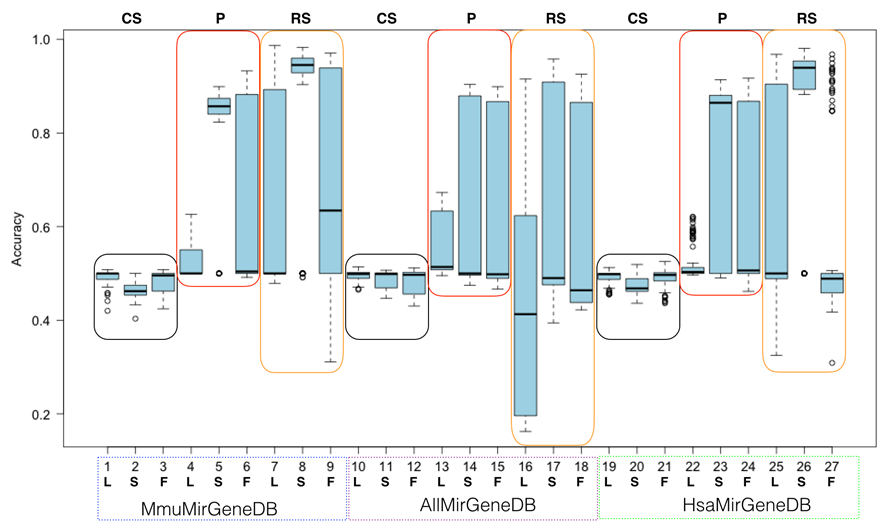


Figure S8: OCC performance based on all combinations of parameters. L, S, F stand for LowVarFS, SlowFS and FastFS, while CS, P and RS refer to ColShuf, Pseudo and RowShuf, respectively. Rectangles indicate the three negative datasets while positive datasets are indicated under the x-axis. Each box plot refers to 100fold MCCV calculations.

Table S1: Selected features for the three *ad hoc* feature selection methods employed in this study. Features in the first group display a low variance and are probably not good for separating data into classes. SlowFS consists of features which are computationally expensive to calculate and are likely to be informative. FastFS contains features which are simple to calculate but not necessarily uninformative. They present the middle ground. For more information about the features and to calculate them please see <http://jlab.iyte.edu.tr/software/mirna>.

| **LowVarFS** | **FastFS** | **SlowFS** |
| --- | --- | --- |
| %AA/hpl | #G((. | dns_z(dc) |
| %AC/hpl | #G..( | dns_z(Q/hpl) |
| %AG/hpl | #GU | dns_p(Q/hpl) |
| %AU/hpl | #G((( | dns_z(Q) |
| %CA/hpl | #G++#G | dns_p(Q) |
| %CC/hpl | #G(.. | dG |
| %CG/hpl | #C(.( | dH |
| %CU/hpl | #C(.. | dS |
| %GA/hpl | #GG | Tm |
| %GC/hpl | #C... | dm |
| %GG/hpl | #GC | dc |
| %GU/hpl | #C..( | dns_z(bpp/hpl) |
| %UA/hpl | #U | dns_z(bpp) |
| %UC/hpl | #GA | dns_p(bpp/hpl) |
| %UG/hpl | #C.(( | dns_p(bpp) |
| %UU/hpl | #CA | dns_z(bpd/hpl) |
| *A(((/hpl | #C.(. | dns_p(bpd/hpl) |
| *A((./hpl | #G++#C | c#Ns |
| *A(.(/hpl | #C((( | dns_z(hpmfe_rf/hpl) |
| *A(../hpl | #C((. | c#N |
| *A.((/sl | #A((( | dns_z(bpd) |
| *A.(./hpl | #CC | c#Ns/sl |
| *A.(./sl | #A++#C | CGsAU/sl |
| *A..(/hpl | #G++#A | lsr(%G)/sl |
| *A.../hpl | #CU | %UA/sl |
| *C(((/hpl | #CG | CUsGA/sl |
| *C((./hpl | #A.(( | lsr(%G-U)/sl |
| *C(.(/hpl | #A.(. | %U++%A/hpl |
| *C(../hpl | #A..( | lsr(%A-U)/sl |
| *C.((/hpl | #A... | c#Gs/hpl |
| *C.(./hpl | #AG | CAsGU/hpl |
| *C..(/hpl | #AA | *C(.(/hpl |
| *C.../hpl | #A((. | #U++#C/sl |
| *G(.(/hpl | #A(.. | *A.((/sl |
| *G(../hpl | #C++#C | *U.../hpl |
| *G.(./hpl | #C | lsr(%G-C)/sl |
| *G..(/hpl | Q | c#C/sl |
| *G.../hpl | clsp | *U(((/sl |
| *N.(./hpl | #AU | #U++#U/sl |
| *U((./hpl | #AC | CUsGA/hpl |
| *U(.(/hpl | #C++#A | hpmfe_rf_I1/sl |
| *U(../hpl | clep | lsr(%U)/sl |
| *U.(./hpl | #A | mwmF/sl |
| *U.(./sl | dme | lsr(%A)/sl |
| *U..(/hpl | #A++#A | GUsAC/sl |
| *U.../hpl | ediv | lsr(%C)/sl |
| adalr/hpl | efq | *C.(./sl |
| dme/hpl | bpd | GAsUC/hpl |
| hpmfe_rf_I2 | hpmfe_rf | *U(((/hpl |
| hpmfe_rf_I3 | efe | CGsUA/hpl |
